# Enhanced Water Resistance of Recycled Newspaper/High Density Polyethylene Composite Laminates via Hydrophobic Modification of Newspaper Laminas

**DOI:** 10.3390/polym13030421

**Published:** 2021-01-28

**Authors:** Binwei Zheng, Weiwei Zhang, Litao Guan, Jin Gu, Dengyun Tu, Chuanshuang Hu

**Affiliations:** College of Materials and Energy, South China Agricultural University, Guangzhou 510642, China; hnzbw@stu.scau.edu.cn (B.Z.); ltguan@scau.edu.cn (L.G.); gujin@scau.edu.cn (J.G.); tudengyun@scau.edu.cn (D.T.)

**Keywords:** recycled newspaper, composite laminates, water resistance, high strength

## Abstract

A high strength recycled newspaper (NP)/high density polyethylene (HDPE) laminated composite was developed using NP laminas as reinforcement and HDPE film as matrix. Herein, NP fiber was modified with stearic acid (SA) to enhance the water resistance of the NP laminas and NP/HDPE composite. The effects of heat treatment and SA concentration on the water resistance and tensile property of NP and composite samples were investigated. The chemical structure of the NP was characterized with X-ray diffractometer, X-ray photoelectron spectroscopy and attenuated total reflectance Fourier transform infrared spectra techniques. The surface and microstructure of the NP sheets were observed by scanning electron microscopy. An expected high-water resistance of NP sheets was achieved due to a chemical bonding that low surface energy SA were grafted onto the modified NP fibers. Results showed that the hydrophobicity of NP increased with increasing the stearic acid concentration. The water resistance of the composite laminates was depended on the hydrophobicity of the NP sheets. The lowest value of 2 h water absorption rate (3.3% ± 0.3%) and thickness swelling rate (2.2% ± 0.4%) of composite were obtained when the SA concentration was 0.15 M. In addition, the introduction of SA can not only enhance the water resistance of the composite laminates, but also reduce the loss of tensile strength in wet conditions, which shows potential in outdoor applications.

## 1. Introduction

Recycled paper has been widely used to fabricate bio-based fuels, fertilizer and high-valued cellulose products in the past decades [1,2,3,4]. As a readily available sheet product with desirable mechanical properties, recycled newspaper has been used to fabricate paper-based composite laminates in recent years [5,6,7]. Excellent mechanical performances of recycled newspaper composite laminates were achieved by forming a compact structure, which could enhance the fiber-fiber bond of paper sheet [8,9]. However, recycled newspaper has poor water resistance due to its hydrophilic nature, resulting in unexpected water uptake behavior of its laminated composite [10]. The strength of paper sheets can be reduced significantly due to the dis-bonding of paper fibers after the absorption of water molecules, leading to a negative effect on mechanical performances of the composites [11]. The poor water resistance greatly limits the applications of this sort of composites, especially for composites with high paper content [12]. Although the composite can be protected laterally, it will inevitably produce scratches or even cracks when they applied outdoors. Water molecules can pass through these defects. Therefore, it is essential to endow hydrophobicity for paper sheets in preparing recycled paper-based composite laminates. 

Some efforts have been made in developing a hydrophobic/superhydrophobic paper sheets to improve the water resistance [13]. It is well known that both high surface roughness with micro/nanoscale structures and low surface energy are beneficial to create a hydrophobic/superhydrophobic coating on paper sheet [14,15,16,17,18,19]. However, some disadvantages still should be considered. On one hand, paper is easily discomposed through physical or chemical damage [20]. On the other hand, superhydrophobic coating are usually weakly adhesive and poorly durable, so the maintenance of their superhydrophobicity is inconvenient and expensive [21]. An alternative method is chemical modification at fiber surface instead of depositing micro-scale coating layer on paper surface. Paper and paperboard are mainly composed of natural cellulosic fibers, which are composed of β-D-glucose polymer with many hydroxyl groups. Hydrophilic surface of cellulosic fibers could be transferred into hydrophobic surface through chemical methods, such as silanization, etherification, and esterification [22].

Stearic acid (SA), a saturated fatty derived from animal and vegetable fats and oils, possesses a long surface energy hydrocarbon chain and active carboxyl group that can form an ester linkage with cellulose hydroxyl groups. With the advantages of low cost, environmental friendliness, and biocompatibility with cellulose fibers, SA has been widely used as a hydrophobic agent for cellulose products in recent years [23,24]. In these cases, cellulose films or paper are usually dipped in organic solvents and stirred vigorously in high temperature to obtain the hydrophobicity, which would damage to the fiber network and even results in dis-bonding of cellulose fibers. The solvent reaction system is regarded as an inefficient method for intact paper sheets modification.

The objective of this work is to improve the water resistance of recycled newspaper enhanced high-density polyethylene composite laminates. Herein, low surface energy stearic acid was firstly deposited on the paper fiber using a solvent evaporation method and then heated to form an ester bonding with a gentle solid-phase reaction method so that the integrity of paper sheets can be maintained. The SA modified paper sheet was stacked as reinforcing layer by using the HDPE film as matrix layer to fabricate composite laminates via hot-pressing. Effects of the modified process and stearic acid concentrations on water resistance of recycled newspaper and the composite laminate were investigated.

## 2. Experimental

### 2.1. Materials

Recycled newspapers (NP), with grammage of 45 g/m^2^ and thickness of 50 μm from Shanghai Securities News, were cut to 200 mm × 200 mm. High-density polyethylene films (HDPE, density 0.95 g/cm^3^, MFR 0.24g/10min at 190 C/2.16 kg, *T*_m_ 119 °C, tensile strength 17 MPa), food packaging film in daily life with the thickness of 12.8 μm, were purchased from local company (Charoen Pokphand Group, Guangzhou, China). Stearic acid (SA) and absolute ethanol (analytical grade) were obtained from Guangzhou Chemical Reagent Factory.

### 2.2. Preparation of Hydrophobic NP

**Scheme 1:**
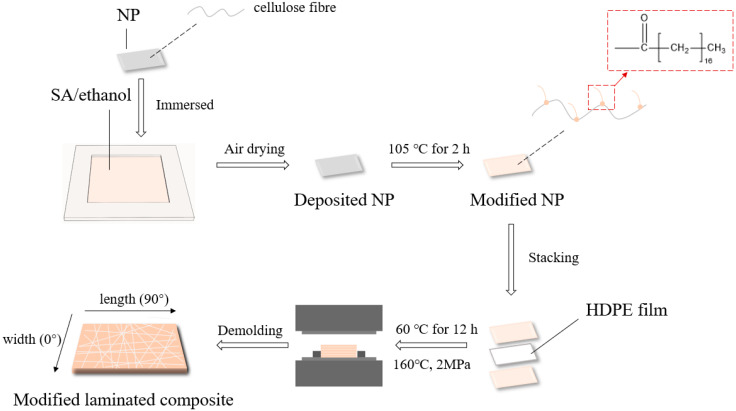
Formation illustration of the hydrophobic NP and composite fabrication process.

The modification process of NP sheets was shown in Scheme 1. The dried SA powder was dissolved in absolute ethanol and then stirred vigorously at 70 °C for 15 min, forming a homogeneous solution. NP sheets were dipped into SA/absolute ethanol solution (0.01, 0.05, 0.1, 0.15, 0.2 M) for 15 min and then dried in air at room temperature, which was named as deposited NP. The SA-deposited NP sheets were further heated in an oven at 105 °C for 2 h and then cooled down to room temperature, which was called modified NP.

### 2.3. Composite Fabrication

Laminates were prepared by stacking NP sheets and HDPE films alternately with the same direction. Herein, each NP sheet was stacked with one layer of the HDPE film to control paper content of the laminates at 78.5 wt%. In total, 20 layers of newspaper and 20 layers of HDPE film were used. The prepared laminates were oven-dried at 50 °C for 24 h and then transferred to the pre-heated hot press machine (BY302X2/2 150T, Suzhou New Cooperative, Suzhou, China). A steel bar frame as a thickness gauge was used to control the thickness of the composites at 1 mm. Poly-tetrafluoroethylene (PTFE) films were used as demolding layer to facilitate the demolding process. Composites were fabricated by setting the pressing pressure and the heating temperature at 2.0 MPa and 160 °C, respectively, holding for 25 min to ensure a sufficient compaction and infiltration. After the hot-pressing procedure was completed, the hot panel was quickly cooled down to room temperature by using circulation water cooling system. Finally, the pressure was released, and the composite laminates (NP/HDPE composite) was removed. As NP is a typical anisotropic material with different mechanical properties at fiber direction (0°, wide direction of newspaper, 52.0 MPa of tensile strength) and perpendicular fiber direction (90°, length direction of newspaper, 12.9 MPa of tensile strength). Specimens for property testing were cut from the composites along parallel directions by using a mold cutter (GT7016HA, Gotech testing machine Co., Ltd, Dongguan, China).

### 2.4. Characterizations

The phase structure of the NP samples was characterized by X-ray diffractometer (XRD) (D8 Advance, Bruker, Germany). The patterns were recorded in the region of 2θ from 4° to 70° with a scanning speed of 10°/min at 40 kV and 40 mA (copper Kα radiation λ = 0.154 nm). Attenuated Total Reflectance Fourier transform infrared spectra (ATR-FTIR) (TENSOR27, Bruker, Germany) was used to analyze functional groups of the NP and composites samples in the range of 4000–600 cm^−1^ with 32 scans and a resolution of 4 cm^−1^. Chemical elements of the NP samples surface were obtained by using X-ray photoelectron spectroscopy (XPS) (Thermo EscaLAB 250Xi, Shanghai, China), with an Al X-radiation (Kα, hv = 1486.8 eV). All of the binding energies were corrected based on the C1s peak at 284.8 eV. The microstructure of pristine, deposited and modified NP samples were observed by scanning electron microscopy (SEM) (EVO-18, Zeiss, Jena, Germany) with an acceleration voltage of 10 kV. All of the samples were coated with a thin carbon conductive coating via sputter deposition before testing.

### 2.5. Physical and Mechanical Properties Testing

The hydrophobicity of the NP sheets was assessed by water contact angle (WCA) and water absorption rate according to ASTM D 724 and GB/T 461.3, respectively. WCA was performed with an optical Contact Angle Meter (DSA100, Kruss, Germany). A water droplet size of 5 μL was placed on NP sample surface. Water absorption rate of the NP sheets with the dimension of 100 mm × 100 mm was calculated by measuring the mass of the sample before and after immersing in a tank full of deionized water. Ten repeats of WCA and water absorption rate of each NP sample were carried out.

2 h-water absorption and thickness swelling tests were conducted to evaluate the water uptake behavior of composite laminates in accordance with ASTM D 570. The dimension of the sample was 76.2 × 25.4 mm^2^. Samples were weighed and thickness measured before and after stored in distilled water. Three test bars were conducted for every composite sample with different hydrophobic modification conditions.

Tensile property of the pristine and hydrophobic modification NP sheets in dry and wet conditions were measured by using an electromechanical universal testing machine (CMT5504, Shenzhen Rethink Cooperation, China) according to GB/T 12914. The dimension of the sample was 15 mm × 200 mm and the loading speed during the testing was 25 mm/min. All NP samples with 0° direction were tested ten times.

The mechanical performances of composite laminates with 0° direction were also evaluated by investigating the tensile property in dry and wet condition, using an electromechanical universal testing machine (CMT5504, Shenzhen Rethink Cooperation, Shenzhen, China) according to ASTM D 638. For the tensile test, dumbbell specimen size was 165 × 13 mm^2^ and the loading speed was 5 mm/min. Nine replicates of the tests were conducted. 

## 3. Results and Discussion

### 3.1. Morphology and Microstructure

**Figure 1:**
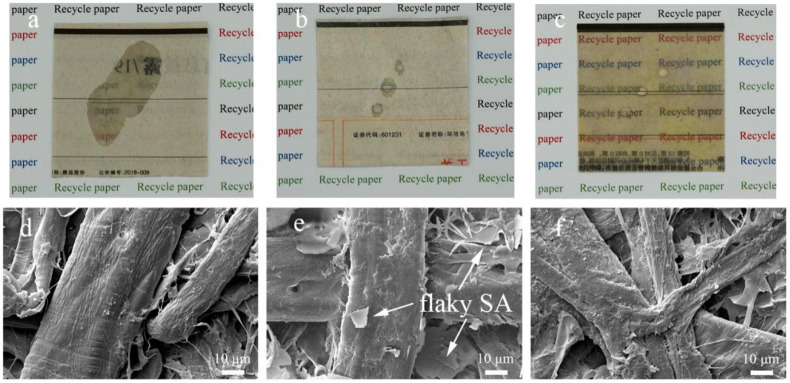
Photographs (**a**–**c**) and SEM (**d**–**f**) images of pristine NP (**a**,**d**), deposited NP (**b**,**e**) and modified NP (**c**,**f**).

The appearance and microstructures of the pristine, the deposited NP and the modified NP samples are shown in Figure 1. Water drop spread on pristine NP surface instantaneously, leaving a large wetting spot. For the deposited NP, it formed a hemisphere firstly but penetrated into NP gradually. For the modified NP, the water beads were hold on the surface steadily. The pristine and deposited NP exhibited opaque while the modified NP turned to semi-transparent. It is known that the opacity of conventional paper is generally caused by a large amount of light scattering due to the different refractive index of cellulose (approximately 1.5) and air (1.0) when light transmits through fiber-air interfaces [25]. The completely exposure of hydrophilic fibers and the porous structure (Figure 1d) in pristine NP result in highly wetting and large amount of light scattering. As can be seen in Figure 1d–f, flaky SA occupied in gaps and pores of fiber network as well as at fiber surface for deposited NP. For modified NP, crystalloid SA disappeared which probably permeated into fiber network due to heat treatment. Li et al. suggested that the transparency of paper sheet can be improved by using transparent agents (with similar refractive index to cellulose) to fill the voids inside paper [26]. It could be inferred that, after heat treatment, SA (refractive index is 1.455) had melted and filled in part of voids inside the NP sheet, resulting in reduced light scattering and semi-transparent appearance of the modified NP. 

**Figure 2:**
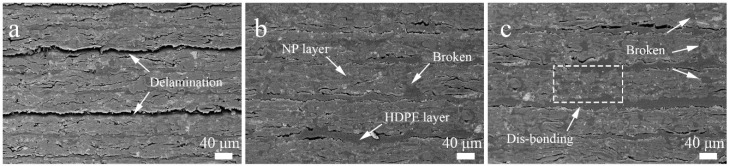
BSEM images of (**a**) unmodified composite; (**b**) 0.05M SA modified composite and (**c**) 0.15M SA modified composite.

The microstructures of unmodified and SA modified composite samples are presented in Figure 2. The multilayer structure of composite samples was obvious that NP sheets and HDPE films were stacked alternately. It was found that HDPE permeated into the NP sheets and occupied the voids, forming a dense composite structure. However, pores and voids could be found clearly because of lacking enough HDPE (only 21.5% HDPE content of the composite) to fully fill. In comparison to unmodified composite samples, the modified composite samples showed intact cross section (Figure 2b,c). It was considered that adding SA could improve the interface bonding between NP fiber and HDPE. Therefore, there was no clear delamination and transverse cracks in modified composite. Since the penetration of HDPE was promoted, the fiber network was broken in modified composite and even dis-bonding in composite modified with high SA concentration (Figure 2c). 

### 3.2. Chemical Structure Characterization of NP

**Figure 3:**
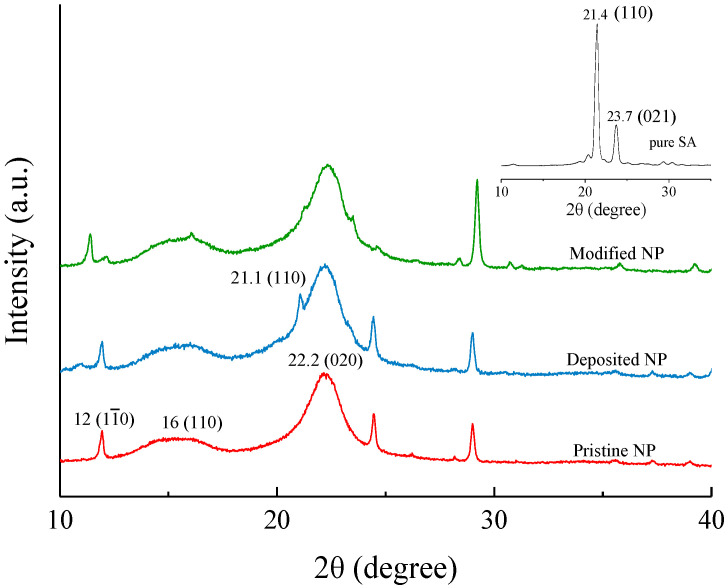
XRD pattern of pristine NP, deposited NP, modified NP and pure SA.

The XRD patterns of pristine NP, SA, deposited NP and modified NP are presented in Figure 3. For all NP samples, three strong diffraction peaks were observed at around 2θ = 12°, 16° and 22.2° which could be assigned to the typical crystalline plane (11(_)0), (110) and (020) of cellulose, respectively [27]. The diffraction patterns of NP samples were not completely consistent with those of pure cellulose because some non-fiber components, such as calcium carbonate filler, ink etc. were found in common waste newspaper [11]. For pure SA sample, the “short spacing” peaks at 2θ = 21.4° and 23.7° were assigned to the (110) and (021) crystallographic planes of the SA respectively due to the presence of the hydrocarbon chain lateral packing order [28,29,30]. It can also be seen that the “short spacing” peak at around 2θ = 21° in deposited NP sample indicates the SA existed as crystal in deposited NP [31,32]. However, the “short spacing” peak was indistinctively found in modified NP. The weak crystalline intensity of SA in modified NP was attributed to its uniform dispersion on the fiber surface. The uniform SA coating contributed to the restriction of hydrocarbon chain mobility so that SA crystal could not be clearly characterized. What’s more, it provided a larger contact area for stearic acid and cellulose fiber to promote interaction.

**Figure 4:**
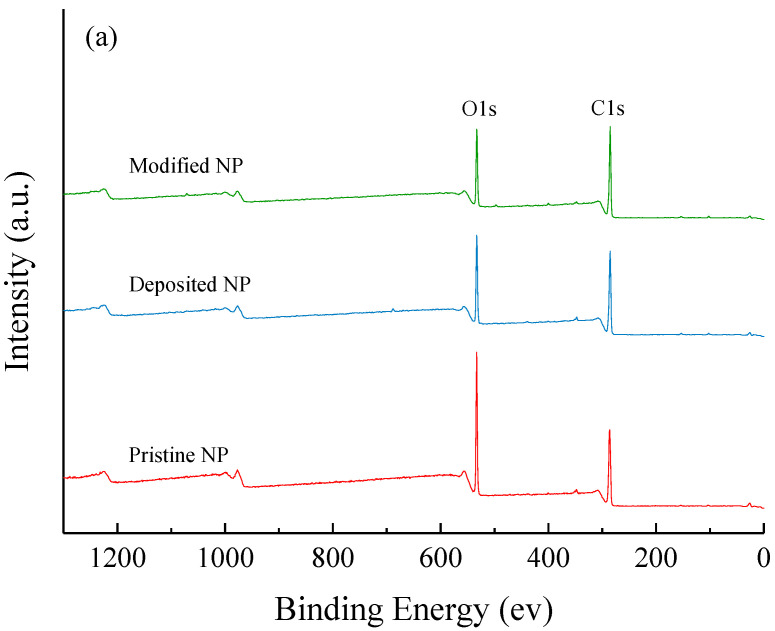

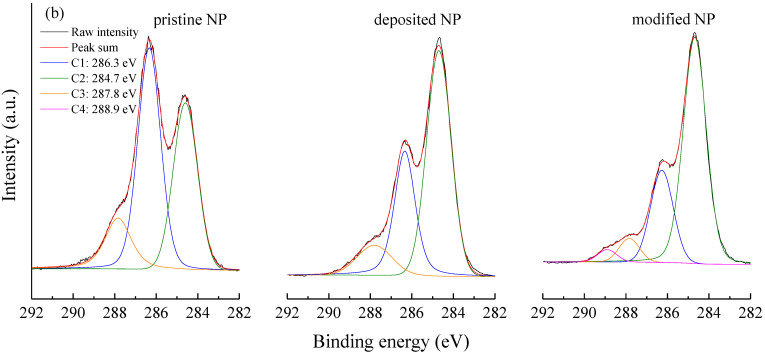
XPS spectra of pristine, deposited and modified NP samples: (**a**) survey spectra and (**b**) deconvolution of the C1s spectra.

The XPS spectra of pristine NP, deposited NP and modified NP samples were presented in Figure 4. All of the NP samples showed the clear C1s (284 eV) and O1s (532 eV) signals (Figure 4a). Three characteristic functional groups C1 (C–O), C2 (C–C), and C3 (C=O) of carbon atoms for cellulose fibers can be found in pristine, deposited, and modified NP samples [16,33]. Compared with pristine NP, both deposited and modified NP samples showed an increase in C/O ratio and C2 (C–C, C–H) ratio (as shown in Table 1), since the coating layer of long carbon chain of SA increased the carbon content [18]. Moreover, the modified NP samples showed additional peaks C4 at 288.9 eV, which was attributed to O–C=O bonds signals from ester linkages. Therefore, it further confirmed that SA was grafted onto the modified NP fiber surface.

**Figure 5:**
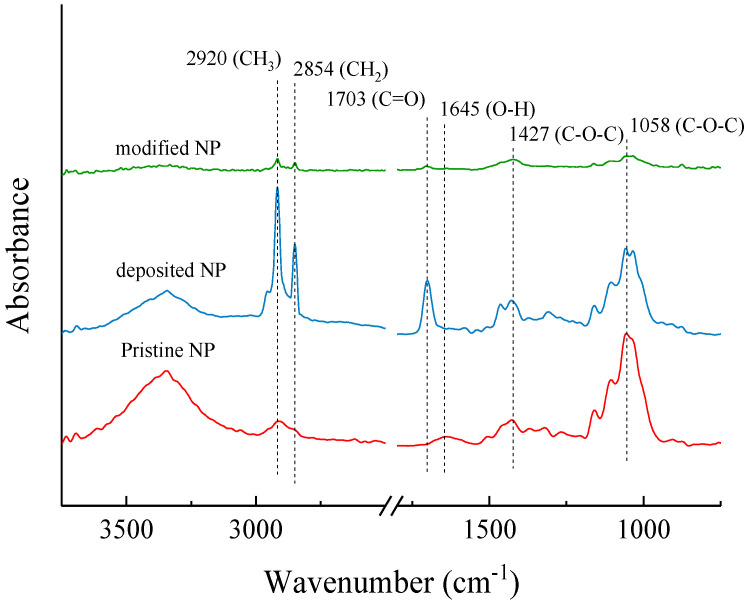
ATR-FTIR spectra of pristine, deposited NP and modified NP.

The ATR-FTIR spectrum was recorded to analyze the chemical bonds of the pristine, deposited and modified NP. As can be seen in Figure 5, the peaks at 2920 cm^−1^ and 2854 cm^−1^ corresponded to the stretching vibration of –CH_3_ and –CH_2_ respectively, which were strongly found in deposited NP due to in-phase stretching groups of long carbon chain of SA while relatively weak in modified NP. It was thought that the stretching vibration of –CH_3_ and –CH_2_ is difficult to be characterized in modified NP because of uniform coating of SA on the fiber surface. The bands at 1427 cm^−1^ and 1058 cm^−1^ were due to the stretching vibration of crystalline regions and C–O–C from cellulose macromolecule of NP fiber. The peak at 1645 cm^−1^, assigned to the bending vibration of O–H from interlayer water molecules, was found obviously in pristine NP but not in the deposited and modified NP. New absorption band at 1703 cm^−1^ in the deposited and modified NP was attributed to the stretching vibration of C=O band after adding SA. Pristine and deposited NP showed broad bands in the region of 3600–3000 cm^−1^ that can be assigned to the stretching vibration of –OH from cellulose. After the heat treatment, both the stretching vibration of –OH from cellulose and C=O from SA were no longer strong, implying that –OH of the NP fiber reacted with –COOH of SA.

### 3.3. Water Resistance and Tensile Property of Modified NP

**Figure 6:**
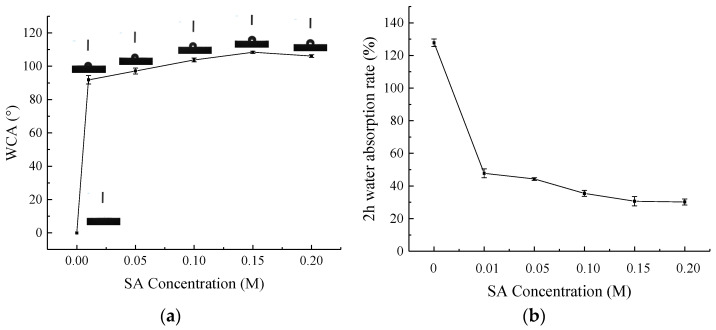
The water contact angle (**a**) and 2 h water absorption rate (**b**) of modified NP with different SA concentrations.

The water contact angle and 2 h-water uptake test were carried out to investigate the water resistance of pristine and modified NP. As shown in Figure 6, all of the SA modified NP sample showed better water resistance than the pristine NP. The water contact angles (WCA) and 2 h water absorption rates of pristine NP samples are 0° and 127.8% ± 2.3%, respectively. High water uptake of the pristine NP could be attributed to the presence of a large number of hydrophilic hydroxyl groups [34]. With increasing SA concentration, the WCA of modified NP samples increased gradually, and the 2 h water absorption rate decreased, since more SA was deposited to react with hydrophilic hydroxyl groups to cause more long carbon chains covering at modified NP surface. When the SA concentration was higher than 0.15 M, the water contact angle and water absorption curve became flat, indicating the SA solution reached the saturate level. After dissolving the SA again (NP sheets were dipped into pure ethanol solution at 70 °C for 15min), the modified NP still exhibited better water resistance (38.1% ± 5.1% of 2 h water absorption rates) than that of the pristine NP. Although the water resistance has been improved, the 2 h water absorption rate was still as high as 30%. As inks and dyes are usually organic compounds, which have a good polarity with SA, they are able to support the SA modification. Therefore, the high-water absorption rate of the modified NP might be attributed to the high amount of inorganic fillers in NP that was added during the paper making. Moreover, the lumen of natural cellulosic fibers could absorb water due to the capillary action.

**Figure 7:**
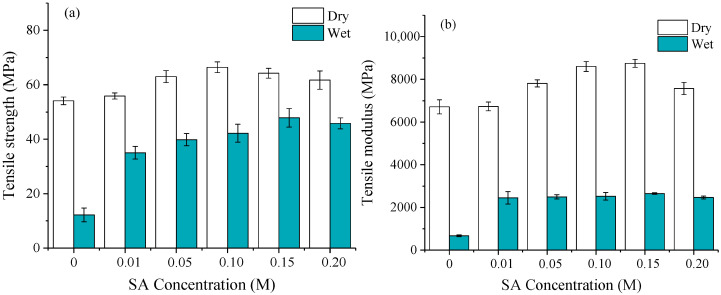
The effect of SA concentration on (**a**) tensile strength and (**b**) tensile modulus of modified NP in dry and wet conditions.

Figure 7 shows the effect of SA concentration on tensile properties of the NP samples in the dry and wet (2 h water immersion) states. Pristine NP exhibited desirable tensile property because of tight fiber network and hydrogen bonds among cellulose fibers. As in Figure 7a, modified NP samples showed higher tensile strength than the pristine NP while the tensile strength of NP increased with increasing the SA concentration. It can be observed in Figure 1c that a certain part of voids inside the modified NP sheets was occupied by SA. It was considered that the filling of SA inside NP sheets can increase the friction between fibers. The tensile strength of all NP samples declined when they were in wet condition because water molecular opened hydrogen bond between fibers [11]. For the pristine NP, it reduced significantly from 54.1 ± 1.4 MPa to 12.2 ± 2.5 MPa. As expected, the modified NP exhibited desirable water resistance due to the uniform coating of hydrophobic carbon chain. So, the tensile strength (in wet) increased from 12.2 ± 2.5 MPa to 47.9 ± 3.4 MPa when the SA concentration increased from 0 to 0.20 M, corresponding to strength loss rate decreased from 78.2% to 25.8%. In Figure 7b, the wet tensile modulus of the modified NP samples showed nearly three times as much as pristine NP while it could not change significantly with increasing the SA concentration.

### 3.4. Water Resistance and Tensile Property of NP/HDPE Composite

**Figure 8:**
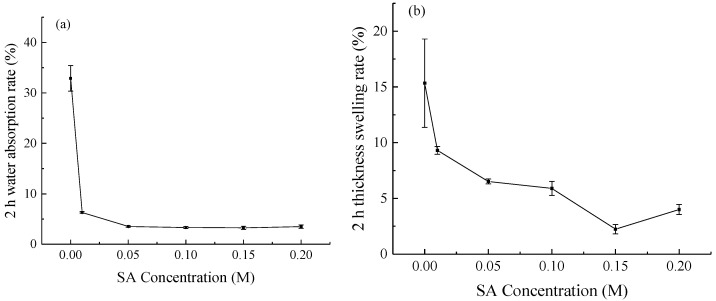
The 2 h water absorption rate (**a**) and 2 h thickness swelling rate (**b**) of NP/HDPE composite with different SA concentrations.

Pristine and modified NP sheets were used to fabricate the NP/HDPE laminated composite via hot-pressing process. The 2 h water absorption rate and thickness swelling rate of composite samples at different SA concentrations were presented in Figure 8. The modified composite samples showed better water resistance than other paper-based composite laminates that 49.3% (with 78.5% paper content) and 10.0% (with 30.0% paper content) water absorption rate were found in literature [10,12]. The 2-h water absorption rate of the composite samples was remarkably lower than that of pristine NP samples, since visible pores and voids in the NP sheets can be filled by HDPE, result in low water uptake [12]. Compared with the unmodified composite, the modified composite (0.01–0.20 M) showed lower water absorption and thickness swelling. As the water adsorption in HDPE could be neglected, the water resistance of the composite laminates is mainly depended on the wettability of NP layer and its porosity. Due to the presence of non-polar long carbon chain of SA, the hydrophobic of the NP sheet was improved. Moreover, a compatible interface between the NP fiber and HDPE was obtained, so paper pores and voids could be fully filled by melted HDPE to block the water transmission. Therefore, with an increasing SA concentration, the water absorption rate fluctuated at around 3%, while the thickness swelling further decreased to 2.2% ± 0.4%.

**Figure 9:**
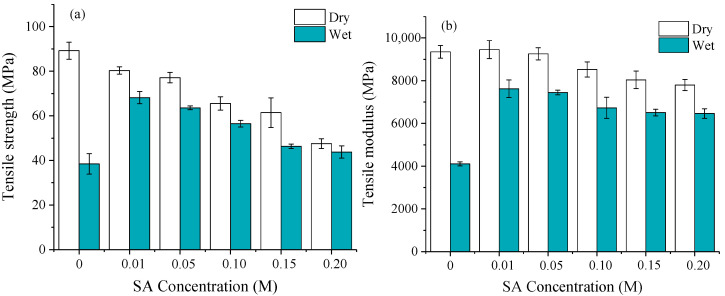
The effect of SA concentration on (**a**) tensile strength and (**b**) tensile modulus of NP/HDPE composite in dry and wet conditions.

The tensile properties of dry and wet NP/HDPE composite samples at different SA concentrations were presented in Figure 9. The dry composite samples showed comparable high tensile strength (from 47–89 MPa) to other paper-based composite laminates (in a range of 58–84 MPa) [7,10,11,35]. The relatively high tensile property was attributed to the dense structure of the composites which can bond the NP fibers tightly and enhance the mechanical interlocking. Results showed that the dry tensile strength and modulus of composites samples fabricated by modified NP decreased with increasing of SA concentration. It was thought that the non-polar long carbon chain coating (grafted from SA) of NP fiber could promote the penetration of non-polar HDPE matrix, leading to the excessive penetration of HDPE in the modified NP layer while 25 min hot-pressing time was suitable for pristine NP layer. As a result, the mechanical interlock of the NP fiber was weakened (Figure 2b,c). Research shows that the mechanical properties of the NP/HDPE composite decreased if the integrity of the NP sheets was destroyed due to excessive penetration of HDPE, confirmed via observing microstructure [36]. Therefore, with an increasing SA concentration, the tensile strength of the composite decreased gradually.

As expected, the modified composite samples exhibited a better water resistance than the unmodified composites samples. Correspondingly, the modified NP showed clearly higher values of tensile strength and modulus than the unmodified NP in wet conditions. Therefore, the introduction of SA can not only improve the water resistance of the composite laminates, but also reduce the loss of tensile strength in wet.

## 4. Conclusions

In this paper, recycled newspaper composite laminates with good water resistance was fabricated using eco-friendly method. NP sheet was modified with stearic acid as reinforcement. The effects of heat treatment and stearic acid concentration on microstructure, water uptake behavior and tensile performance of NP and composite laminates were investigated. The main findings are as follows:

(1) The modified NP showed visible transparency and higher hydrophobicity with increasing the stearic acid concentration in comparison to the pristine and deposited NP.
(2) The water resistance of the composite laminates was significantly improved due to the hydrophobic modification of the NP sheets since lower water uptake and higher wet tensile strength were found in modified composite samples.
(3) The excellent water resistance of NP and its composite laminates were attributed to the esterification reaction between hydrophilic hydroxyl group of NP fiber and carboxyl group of stearic acid.
(4) The introduction of stearic acid can not only improve the water resistance of the composite laminates, but also reduce the loss of tensile strength in wet conditions, which shows potential in outdoor applications. As adding SA can promote the penetration of non-polar HDPE matrix, it was thought that the mechanical property can be improved via altering the holding time.

## Figures and Tables

**Table 1:** Assignment of the XPS C1s peaks.

Samples	Element Concentration (%)		Atomic Ratio	Ratios of C1s Deconvolution			
	C1s	O1s	C/O	C1	C2	C3	C4
				C–O	C–C/C–H	C=O	O–C=O
Pristine NP	65.7	34.3	1.9	48.6	36.6	14.9	0
Deposited NP	72.8	27.2	2.7	31.3	57.5	10.8	0
Modified NP	75.1	24.9	3.0	25.0	66.6	5.7	2.8

## Data Availability

The data presented in this study are available on request from the corresponding author. The data are not publicly available due to our future research needs.
